# Effectiveness of pre-Omicron COVID-19 mRNA vaccines against hospitalizations in infection-naïve children: a case-control study

**DOI:** 10.3389/fped.2024.1387086

**Published:** 2024-11-14

**Authors:** Lung Chang, Ching-Ying Huang, Nan-Chang Chiu, Jin-Yuan Wang, Hsin Chi, Tsung-Ning Daniel Huang

**Affiliations:** ^1^ Department of Pediatric, MacKay Children’s Hospital, Taipei, Taiwan; ^2^ Department of Medicine, Mackay Medical College, New Taipei City, Taiwan

**Keywords:** COVID-19, children, Taiwan, vaccine, effectiveness

## Abstract

This case–control study spanned from July 2022 to September 2023, focusing on the pre-Omicron COVID-19 mRNA vaccine effectiveness against the Omicron variant in children without prior SARS-CoV-2 exposure. We reported that pre-Omicron COVID-19 mRNA vaccines significantly reduced Omicron-induced hospitalizations in infection-naïve Taiwanese children. Our study also highlighted the socioeconomic factors influencing COVID-19 vulnerability among the children population.

## Graphical Abstract

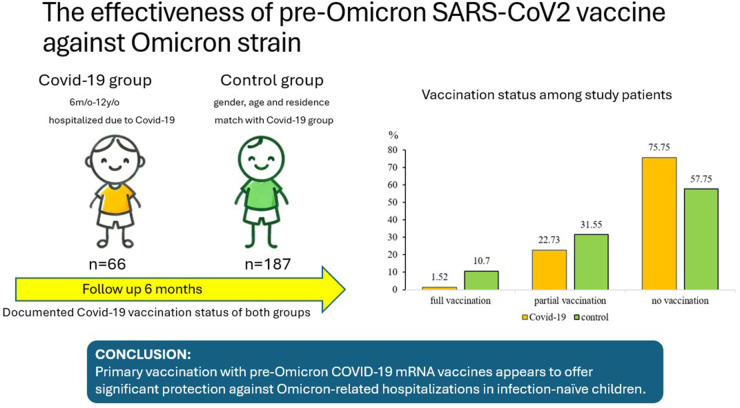

## Introduction

Due to the successful implementation of measures to control the spread of the coronavirus in the early stages of the COVID-19 pandemic, Taiwan remained largely COVID-free until March 2022 (1). However, the country then faced a surge in cases of the Omicron variant. At that time, most of the Taiwanese population had not experienced natural infection but had been vaccinated with SARS-CoV-2 vaccines, such as BNT162b2 and mRNA-1273, which were designed to target earlier strains of the virus, specifically the original Wuhan strain and Alpha variants.

This special situation allowed us to study the effectiveness of pre-Omicron COVID-19 mRNA vaccines against Omicron (e.g., BA.2, BA.5, BA.2.75, and XBB.1)-induced hospitalizations in infection-naïve children.

## Methods

### Study design

We conducted a single-center, matched-pair case–control study involving children aged 6 months to 12 years from July 2022 to April 2023. The study was approved by the institutional review board of MacKay Memorial Hospital (approval number: 22MMHIS057e).

### Study participants

The study enrolled hospitalized pediatric COVID-19 cases as the study group, with cases comprising children admitted to MacKay Children's Hospital, Taipei, confirmed to have COVID-19 by rapid tests or polymerase chain reaction (PCR). The control group was selected based on specific matching criteria: same age, identical gender, similar place of residence, and no history of COVID-19 hospitalization.

### Data collection

Demographic characteristics of study participants, including age, sex, body weight, underlying disease, vaccination status, vitamin D supplement use, and family income, were collected. For controls, a 6-month follow-up call was conducted after recruitment to exclude any with subsequent COVID-19-related hospitalizations. Monthly telephone follow-ups were conducted with all enrolled patients to monitor their health status, and any notable medical history or confirmed COVID-19 cases were documented.

### Matching criteria and case–control ratio

Controls were matched to cases based on age, gender, and place of residence. The case–control ratio varied between 1:1 and 1:3 due to attrition. While an ideal 1:3 ratio was preferred, a 1:2 or 1:1 case–control ratio was also deemed acceptable for analysis.

### Vaccination definitions

Vaccination status was categorized as follows. Full vaccination, receiving two mRNA vaccine doses 28 days apart according to the standard protocol (or three doses for BNT162b2 among children <5 years old). Partial vaccination, receiving at least one dose but not completing the standard two or three doses of COVID-19 vaccines.

### Statistical analysis

Data were analyzed using conditional logistic regression, with vaccine effectiveness (VE) estimated as 1 minus the odds ratio (OR). Statistical significance was set at *p*-values ≤ 0.05. All data analyses were performed using Stata software (v11.0; StataCorp, College Station, TX, USA).

## Results

### Study population

**Figure 1:**
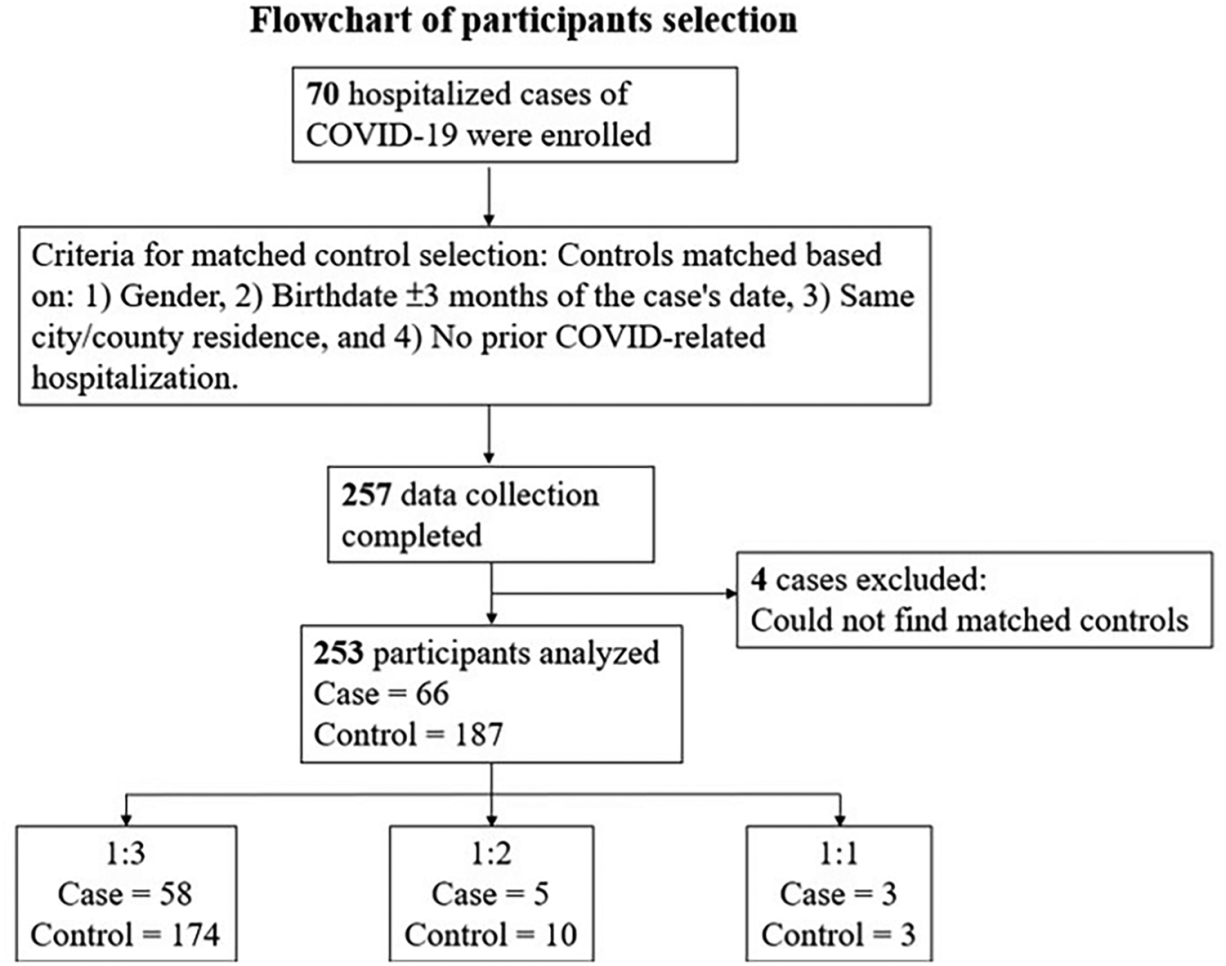
Flowchart of participants selection.

In total, 253 children participated: 66 cases and 187 controls (Figure 1). Children in the case group were admitted to the hospital with a variety of diagnoses, including rhabdomyolysis, pneumonia, and encephalitis, among others. Seventeen patients (25.8%) in the case group reported symptoms of long COVID syndrome, with malaise and coughing being the most common.

### Vaccine effectiveness

**Figure 2:**
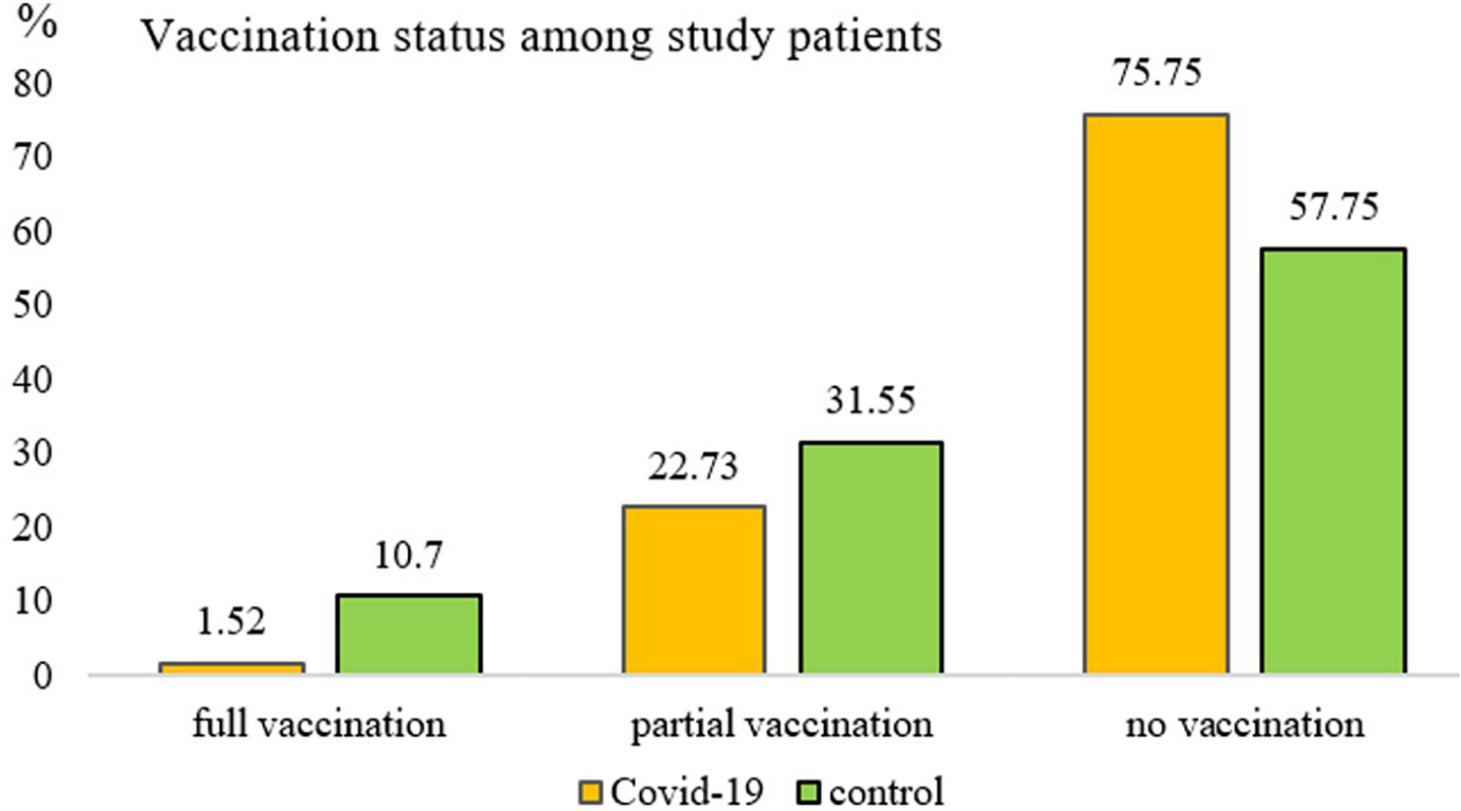
Vaccination status among study patients.

None of the children in the control group were hospitalized due to COVID-19 during the 6-month study period. Among the controls, 108 were unvaccinated, 20 were fully vaccinated, and 59 were partially vaccinated. The average time from the first dose of the vaccine to the time of participant recruitment was 2.3 months, ranging from 0.1 to 8.4 months. Full vaccination considerably reduced COVID-related hospitalization risks (OR, 0.10; 95% CI, 0.01–0.76), as did partial vaccination (OR, 0.45; 95% CI, 0.22–0.93) (Figure 2).

### Socioeconomic factors

**Figure 3:**
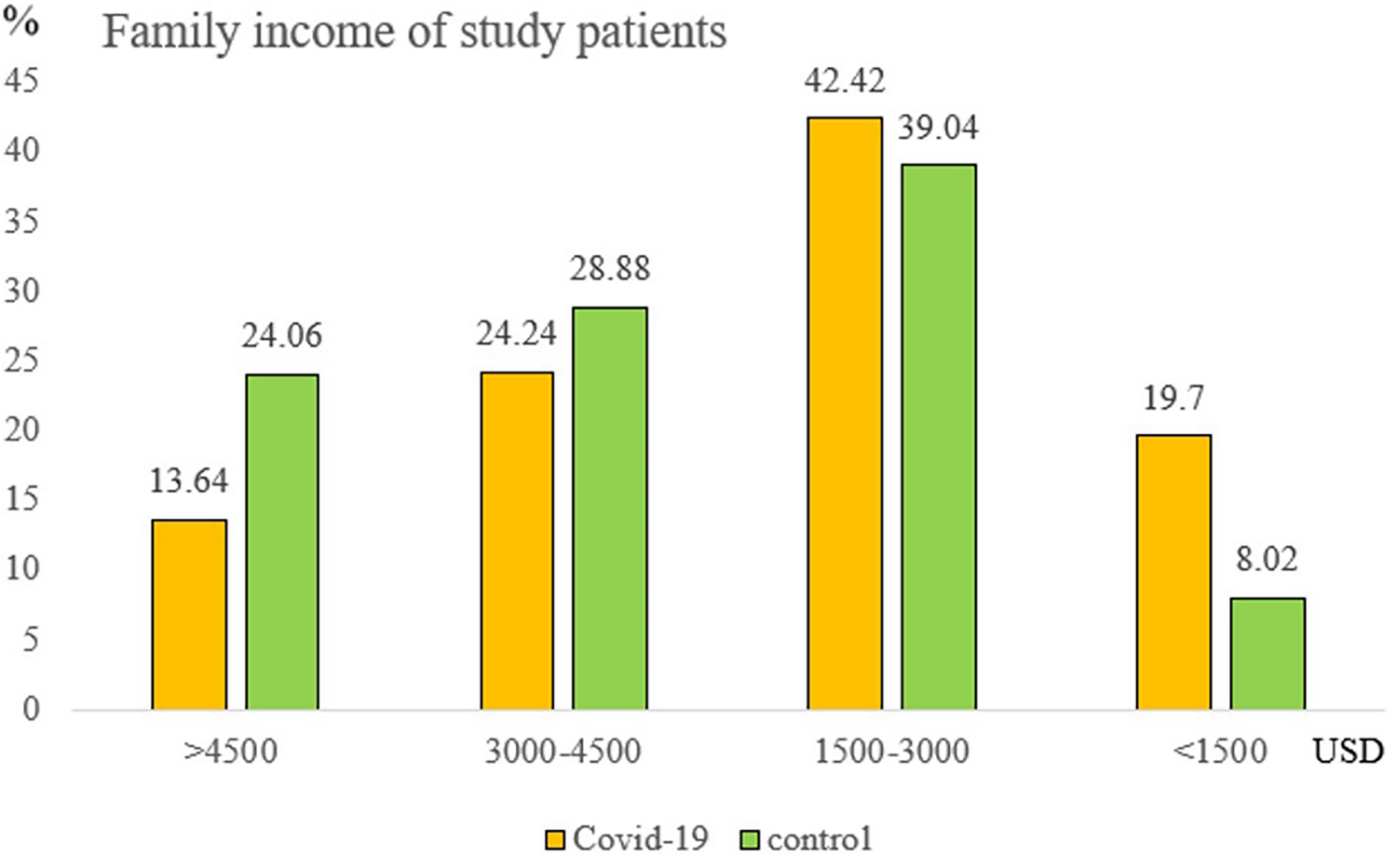
Family income of study patients.

A noteworthy finding was family income's protective effect. Compared to incomes <1,500 USD/month, the hospitalization decrease rate was 75%, 60%, and 50% for incomes >4,500 USD, 3,000–4,500 USD, and 1,500–3,000 USD/month, respectively (Figure 3). After multivariate adjustments, both full vaccination and higher family incomes remained significant protectors against COVID-related hospitalizations (*p*-value = 0.002 and *p*-value = 0.017, respectively). The patient’s age, sex, body weight, underlying disease, and vitamin D level showed no significant difference (Supplementary Table S1).

## Discussion

Since the global COVID-19 pandemic began in 2019, the development of vaccines against SARS-CoV-2 has accelerated, leading to approvals starting in 2020. However, the effectiveness of mRNA vaccines has diminished as new variants of SARS-CoV-2 have emerged (2). The data on vaccine effectiveness in providing cross-protection against different SARS-CoV-2 variants were inconclusive, likely due to the high seroprevalence in the general population (3, 4).

This study reaffirms that pre-Omicron COVID-19 mRNA vaccines (BNT162b2 and mRNA-1273) effectively prevent Omicron-related pediatric hospitalizations in Taiwan, most of the population did not have COVID-19 before. The result suggests that mRNA vaccines may provide some level of protection against different variants of SARS-CoV-2. However, the Advisory Committee on Immunization Practices (ACIP) recommends prioritizing booster doses for children, particularly those with underlying health conditions and comorbidities, to strengthen protection against emerging variants of SARS-CoV-2.

Our study also underlines the socioeconomic influence on COVID-19 vulnerability, echoing other research findings on the disproportionate impact of COVID-19 on economically disadvantaged populations (5). This phenomenon may be attributed to several unmeasured factors, such as healthcare access or lifestyle habits, which could have influenced the results and may be linked to economic status.

However, our study has some limitations: the relatively small sample size given its single-center nature, presumptive uninfected status without serological evidence, potential misclassification due to test inaccuracies, and an inability to account for waning vaccine protection (6). Few children of the control group (7.5%) got vaccinated during the 6-month follow-up, possibly skewing partial vaccination VE estimates. Additionally, undetected or asymptomatic SARS-CoV-2 infections may have contributed to immunity against COVID-19-related hospitalizations.

In conclusion, primary vaccination with pre-Omicron COVID-19 mRNA vaccines appears to offer significant protection against Omicron-related hospitalizations in infection-naïve children. Future public health measures should consider socioeconomic factors when addressing pediatric hospitalizations related to COVID-19.

## Data Availability

The original contributions presented in the study are included in the article/Supplementary Material, further inquiries can be directed to the corresponding author.
