# Supplementary material for: Effectiveness of pre-Omicron COVID-19 mRNA vaccines against hospitalizations in infection-naïve children: a case-control study
Source: Front Pediatr. 2024 Nov 14;12:1387086. doi: 10.3389/fped.2024.1387086 (PMC11602272; doi:10.3389/fped.2024.1387086)
Supplement: Supplementary file 1 [file Table1.pdf]

Supplementary Table 1. Characteristics Of Study Participants And Odds Ratios Of The Factors Against COVID-19 Hospitalizations

	Cases (N=66), No. (%)	Controls (N=187), No. (%)	OR (95% CI)	Adjusted OR (95% CI)
**Demographic Characteristics of Study Participants**				
Year of enrollment				*P*-value = 0.510
2022	55 (83.33)	162 (86.63)	NA	NA
2023	11 (16.67)	25 (13.37)	NA	NA
Age				*P*-value = 0.879
6m-5yrs	59 (89.39)	169 (90.37)	NA	NA
6-12yrs	7 (10.61)	18 (9.63)	NA	NA
Sex				*P*-value = 0.927
Female	23 (34.85)	64 (34.22)	NA	NA
Male	43 (65.15)	123 (65.78)	NA	NA
**Vaccination Status**				*****P*-value = 0.002**
Full vaccination	1 (1.52)	20 (10.70)	**0.098 *(0.013-0.757)**	**0.114 *(0.014-0.940)**
Partial vaccination	15 (22.73)	59 (31.55)	**0.447 *(0.216-0.927)**	0.690 (0.294-1.617)
No vaccination	50 (75.75)	108 (57.75)	NA	NA
**Body weight**				*P*-value = 0.253
Overweight (BMI>85%)	15 (22.73)	24 (12.83)	1.578 (0.721-3.452)	1.362 (0.560-3.312)
**Vitamin D supplement (in the past month)**				*P*-value = 0.163
Regular	15 (22.73)	61 (32.62)	0.611 (0.307-1.220)	0.974 (0.429-3.310)
**Underlying diseases**				
Asthma	4 (6.06)	17 (9.09)	0.683 (0.227-2.061)	1.151 (0.285-4.658)
History of preterm birth	6 (9.09)	22 (11.76)	0.766 (0.292-2.007)	0.829 (0.288-2.391)
History of hospitalization for respiratory diseases	9 (13.64)	33 (17.65)	0.738 (0.336-1.622)	0.542 (0.188-1.559)
**Family incomes (monthly)**				****P*-value = 0.017**
>4,500 USD	9 (13.64)	45 (24.06)	**0.249 **(0.089-0.699)**	**0.205 *(0.060-0.698)**
3,000-4,500 USD	16 (24.24)	54 (28.88)	**0.402 *(0.169-0.957)**	**0.033 *(0.105-0.912)**
1,500-3,000 USD	28 (42.42)	73 (39.04)	0.500 (0.214-1.167)	0.347 (0.119-1.010)

<1,500 USD	13(19.70)	15(8.02)	NA	NA

Note: *$p < 0.05$, **$p < 0.01$.
